# Identification of putative secretory and membranous proteins of *Chlamydia trachomatis* using bioinformatics tools

**DOI:** 10.1186/1471-2334-14-S3-P6

**Published:** 2014-05-27

**Authors:** Prashant K Mishra, Renu Pandey, Uma Chaudhry, Daman Saluja

**Affiliations:** 1 Dr. B. R. Ambedkar Center for Biomedical Research, University of Delhi, Delhi- 110007, India; 2 Bhaskaracharya College of Applied sciences, University of Delhi, Delhi-110075, India

## Background

Intuitive understanding of hypothetical genes’s signal peptide, transmembrane helix (TH) domain and LipoP box of any pathogenic microorganisms is a prerequisite for the rational development of drugs and vaccine candidates. A protein harboring these sequences may serve as receptor and conveyer for various biostimulations generated during host-pathogen interaction. Identification and annotation of these proteins may help in designing of novel drug or developing new vaccine candidates to control CT infections.

## Methods

We have retrieved whole sequence of CT serovar D/UW-3/CX from NCBI database. To identify secreted proteins, TH domain and lipoP box, we have exploited the following online softwares: sigpred (http://bmbpcu36.leeds.ac.uk/cgi bin/sig_pred), siglanlP4.0 (http://www.cbs.dtu.dk/services/SignalP), TMpred (http://www.ch.embnet.org/software/TMPRED_form.html), TMHMM (http://www.cbs.dtu.dk/services/TMHMM/) and LipoP box (http://www.cbs.dtu.dk/services/LipoP/).

## Results

The present study focused on the 336 HPs of CT to find out signal peptide, TH domain and lipoP box. Out of 336 HPs. Forty proteins have lipoP box and five proteins (CT149, CT605, CT623, CT823 and CT858) have signal peptide as well as TH domain. Additionally, among five, four proteins (CT149, CT623, CT823 and CT858) also have lipoP box. Harboring TH domain sequences suggests that they may have either membrane spanning, extracellular or intracellular domain which may help in host-pathogen interactions and/or signal transduction. It is also possible that these proteins may be secreted and thereby induces host immune responses but it will need further validation.

## Conclusion

We consider that CT149, CT605, CT623, CT823 and CT858 may be secreted outside the pathogen and should be explored further to understand the pathogenesis of CT.

